# Green and Sustainable Chemistry Approaches on Azide‐Based Click Reactions in Polymer Science

**DOI:** 10.1002/marc.202500171

**Published:** 2025-08-06

**Authors:** Hatice Mutlu, Bercis Pektas, C. Remzi Becer, Azra Kocaarslan

**Affiliations:** ^1^ Institut de Science des Matériaux de Mulhouse UMR 7361 CNRS/Université de Haute Alsace Mulhouse France; ^2^ Department of Polymer Chemistry, Technical Polymer Chemistry Rheinland‐Pfälzische Technische Universität Kaiserslautern‐Landau Kaiserslautern Germany; ^3^ Polymer Chemistry Leibniz‐Institut für Verbundwerkstoffe GmbH (IVW) Kaiserslautern Germany; ^4^ Department of Chemistry University of Warwick Coventry Coventry UK; ^5^ Institute of Nanotechnology (INT) Karlsruhe Institute of Technology Eggenstein‐Leopoldshafen Germany; ^6^ Institute of Functional Interfaces (IFG) Karlsruhe Institute of Technology Eggenstein‐Leopoldshafen Germany

**Keywords:** azide‐alkyne cycloaddition, click chemistry, click polymerization, copper‐catalysed azide‐alkyne cycloaddition, metal‐free click chemistry, sustainable chemistry

## Abstract

Click Chemistry, particularly the azide‐alkyne cycloaddition (AAC) reaction, has revolutionized polymer chemistry, enabling precise and efficient synthesis of advanced functional materials. With its high regioselectivity, mild reaction conditions, and versatility, AAC reactions align closely with the principles of Green and Sustainable Chemistry. However, the core principles of Click Chemistry, particularly its compatibility with Green Chemistry ideals—such as reduced waste, high atom economy, and mild reaction conditions—remain insufficiently emphasized in the context of polymer chemistry. The review evaluates current limitations in AAC—particularly the challenges associated with hazardous azide reagents and reliance on non‐renewable resources—and explores innovative solutions, including greener catalysts, solvent‐free systems, and the incorporation of renewable feedstocks. Additionally, the review presents a comparison of activation methods, spanning thermal, catalytic, metal‐free, and strain‐promoted pathways, to highlight their respective advantages and trade‐offs in sustainability. Practical applications of AAC in polymer design are discussed, showcasing its role in creating materials with tailored properties such as thermal stability, bioactivity, and electronic functionality. This analysis provides a roadmap for future research to optimize AAC for sustainability without compromising its effectiveness in materials design.

## Graphical abstract

Click Chemistry, particularly the azide‐alkyne cycloaddition (AAC) reaction, has revolutionized polymer chemistry, enabling precise and efficient synthesis of advanced functional materials. With its high regioselectivity, mild reaction conditions, and versatility, AAC reactions align closely with the principles of Green and Sustainable Chemistry. This mini‐review has highlighted the sustainability potential and challenges of AAC‐based polymerization, with a focus on catalyst choice, reaction conditions, solvent use, and end‐of‐life strategies for materials.

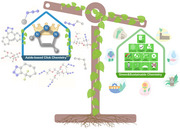

## 1 Introduction

**FIGURE 1:**
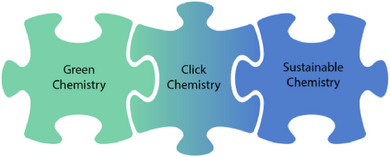
Interconnection of Green Chemistry, Click Chemistry, and Sustainable Chemistry. A representation of how Click Chemistry acts as a bridge between Green Chemistry principles (efficiency, minimal waste) and Sustainable Chemistry goals (environmental impact mitigation) to advance eco‐friendly chemical practices.

The urge to understand the mechanisms of Nature has led to the demand for the versatile use of synthetic chemistry toolbox. In this regard, synthetic chemistry plays a pivotal role in bridging the gap between scientific inquiry and practical innovation in a very wide range, from mimicking small molecules in biological systems to producing durable materials. Therefore, understanding Nature involves not only grasping its chemistry but also minimizing environmental impact through sustainable practices. Green and Sustainable Chemistries all in one serve as a cornerstone, guiding scientists and researchers toward environmentally friendly solutions. In that sense, click chemistry inherently has common grounds by fulfilling Green and Sustainable Chemistry features (Figure 1).

The catchy term “Click Chemistry” was introduced in the field of organic by B. Sharples in 2001 to describe reactions with certain characteristics, emphasizing that “…all searches must be restricted to molecules that are easy to make.” Based on this definition, a reaction must fulfil certain criteria in order to be defined as Click Chemistry. In other words, Click Chemistry relies on modular, high‐yielding reactions that are simple to perform, produce minimal by‐products, and proceed under mild conditions. If purification is required, it should be accomplished through nonchromatographic techniques like crystallization or distillation, ensuring the product remains stable under physiological conditions.

Since the first Huisgen reaction was reported numerous studies have been published on azide‐based organic reactions [1]. In 2004, several pivotal studies introduced the copper‐catalysed azide‐alkyne cycloaddition (CuAAC) to the fields of medicinal chemistry, polymer chemistry, and material science [2, 3, 4]. The CuAAC reaction has been instrumental in advancing polymer chemistry, enabling the precise and efficient synthesis of complex polymer architectures including polymerization, functionalization, block copolymer formation, and crosslink formation. Its versatility and reliability have made the CuAAC a pillar in the development of novel materials and functional polymers. In particular, the work by Sharpless, Fokin, Voit, Hawker and coworkers in 2004 marked a significant milestone in polymer chemistry, showcasing the potential of CuAAC in creating highly efficient dendrimer structures [5]. This foundational study paved the way for further research into using CuAAC to modify and functionalize polymers. Reflecting its transformative impact, Click Chemistry was honoured with the prestigious 2022 Nobel Prize in Chemistry, awarded to Carolyn R. Bertozzi, Morten Meldal, and K. Barry Sharpless [6].

**FIGURE 2:**
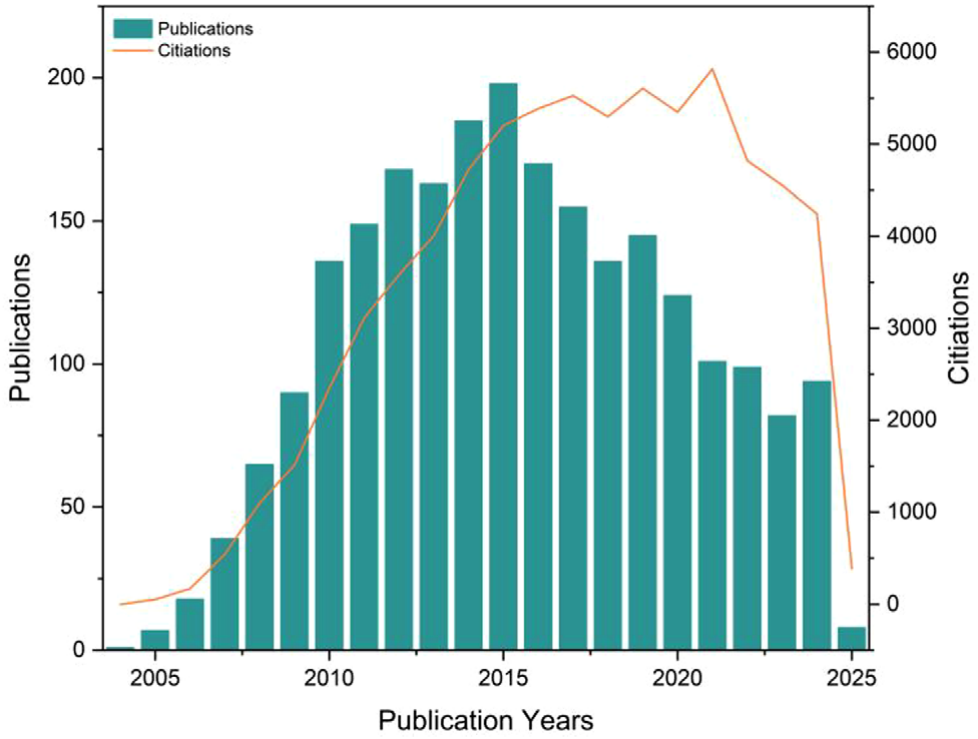
The research trend on azide‐alkyne click polymer chemistry from 2002 to 2025, based on data from the Web of Science (WoS) Core Collection (updated on February 2, 2025), includes 2 333 publications. The publication and citation distribution are represented as the number of publications (left y‐axis) versus citation records (right y‐axis). The data was retrieved using the following search criteria: Topic—azide‐alkyne click polymer chemistry; Document Type—article paper.

In other words, the AAC reaction become a hallmark of ‘Click Chemistry’ and has revolutionized from polymer chemistry to medicinal chemistry due to its robustness, regioselectivity, and tolerance to a wide range of functional groups. As of February 2025, the AAC reaction has been extensively studied, with over 2000 publications indexed in Web of Science (Figure 2). These numbers reflect the significant impact of AAC across various fields, including polymer chemistry, materials science, and bioconjugation.

On the one hand, while azide chemistry offers substantial benefits, it also presents significant challenges for the polymer industry, especially as sustainability requirements continue to evolve. In other words, the limitations of traditional azide chemistry have highlighted the need for more sustainable, efficient, and safer chemical processes in polymer synthesis. On the other hand, azide chemistry is promising due to its electron‐rich nature with extensive application in very diverse synthetic approaches from small molecule synthesis to macromolecular design [7]. The high reactivity of azide moiety and versatility have made it a key building block for the functionalization and modification of polymeric materials. For instance, azido‐functionalized monomers and polymers serve as valuable precursors for post‐polymerization modifications, enabling the introduction of diverse functional groups with high efficiency [8, 9]. The π bond in the azido group can be easily polarized, facilitating strong exothermic dissociation reactions that release reactive nitrene species and molecular nitrogen—an attribute exploited in the design of high‐energy‐density materials, including energetic polymers and propellants. Moreover, organic azides are key synthetic intermediates for most triazole‐ and tetrazole‐containing polymers, which inherently enhance thermal stability and mechanical properties in the resulting polymer while enriching the nitrogen content [10]. Overall, the AAC reaction has enabled the efficient construction of well‐defined polymer architectures, surface modifications, and the development of advanced materials with tailored properties for applications in coatings, drug delivery systems, and nanotechnology [11, 12, 13].

Hence, in this review, we evaluate the potential of azide‐based click chemistry in polymer science, focusing on its sustainability and environmental impact. In other words, the review evaluates how this versatile technique, which has been popular in the field of polymer chemistry and materials science, can be optimized by exploring greener alternatives and methodologies (such as using less hazardous solvents, catalysts, or solvent‐free reactions) to address the inherent limitations of traditional azide chemistry and could help minimize the negative impact while still maintaining the effectiveness of the chemistry. Furthermore, the evolution of more sustainable practices, including those that require less energy or involve renewable feedstocks, is evaluated as a way to improve the ecological footprint of polymerization processes.

## 2 Green Chemistry

Synthetic chemistry has significantly impacted diverse industries by supplying valuable materials—from everyday products to advanced compounds like polymers, liquid crystals, and nanoparticles. However, while these innovations offer numerous benefits, the expansion of production has also introduced challenges, including environmental pollution, the occurrence of ecological disasters, and unsafe working conditions.

The concept of *Green Chemistry* is an emerging field that was established in the early 1990s and defined as a “design of chemical products and processes to reduce or eliminate the use and generation of hazardous substances.” by Paul Anastas et al. [14]. This goal can be achieved by designing more resource‐efficient and inherently safer molecules, materials, products, and processes [15]. The core principle of green chemistry emphasizes that chemical designers have the responsibility of anticipating the long‐term impacts of their chemical agents once they are used in the chemical process. The framework of Green Chemistry can be summarized into three key points: i) it encompasses design considerations throughout all stages of the chemical life‐cycle, ii) it aims to modify the fundamental characteristics of chemical products and processes thus to minimize their inherent risks, iii) Green Chemistry functions as a unified system of principles or design criteria.

**FIGURE 3:**
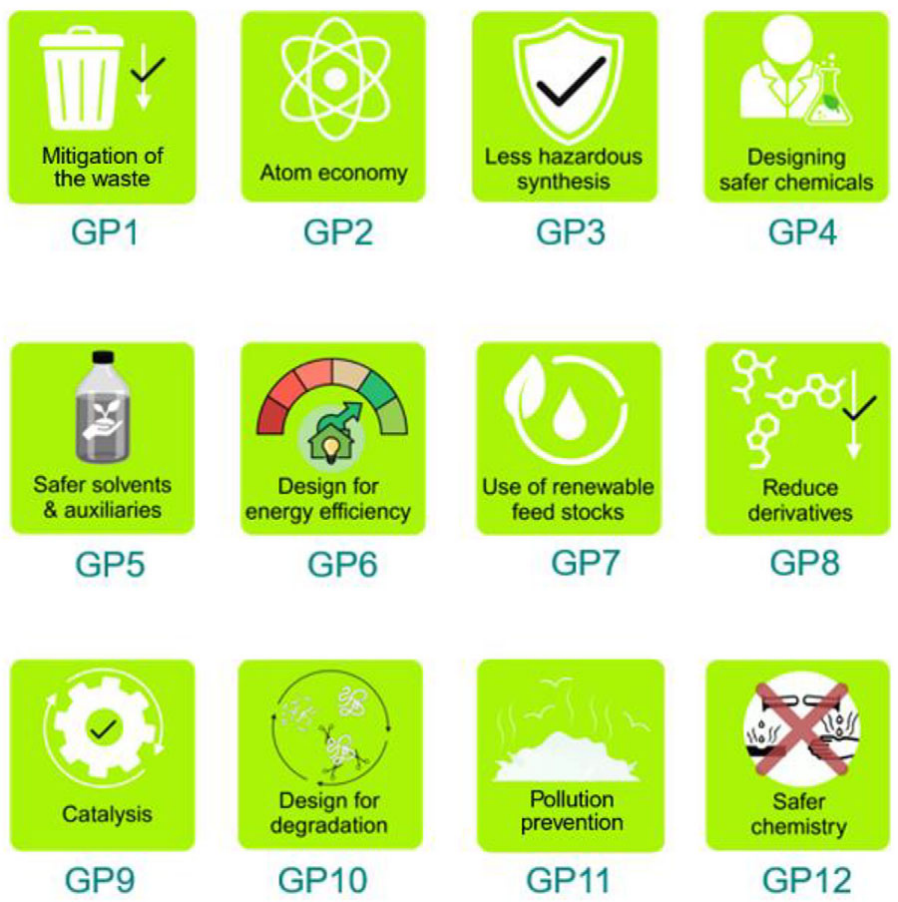
A set of icons illustrating the core 12 principles of Green Chemistry for sustainable innovation.

In this regard, the twelve Green Chemistry principles (GP) provide the framework for sustainable design (Figure 3).

GP1. Prevention
GP2. Atom economy
GP3. Less hazardous chemical synthesis
GP4. Designing safer chemicals
GP5. Safer solvents and auxiliaries
GP6. Design for energy efficiency
GP7. Use of renewable feedstocks
GP8. Reduce derivatives
GP9. Catalysis
GP10. Design for degradation
GP11. Real‐time analysis for pollution prevention
GP12. Inherently safer chemistry for accident prevention

**FIGURE 4:**
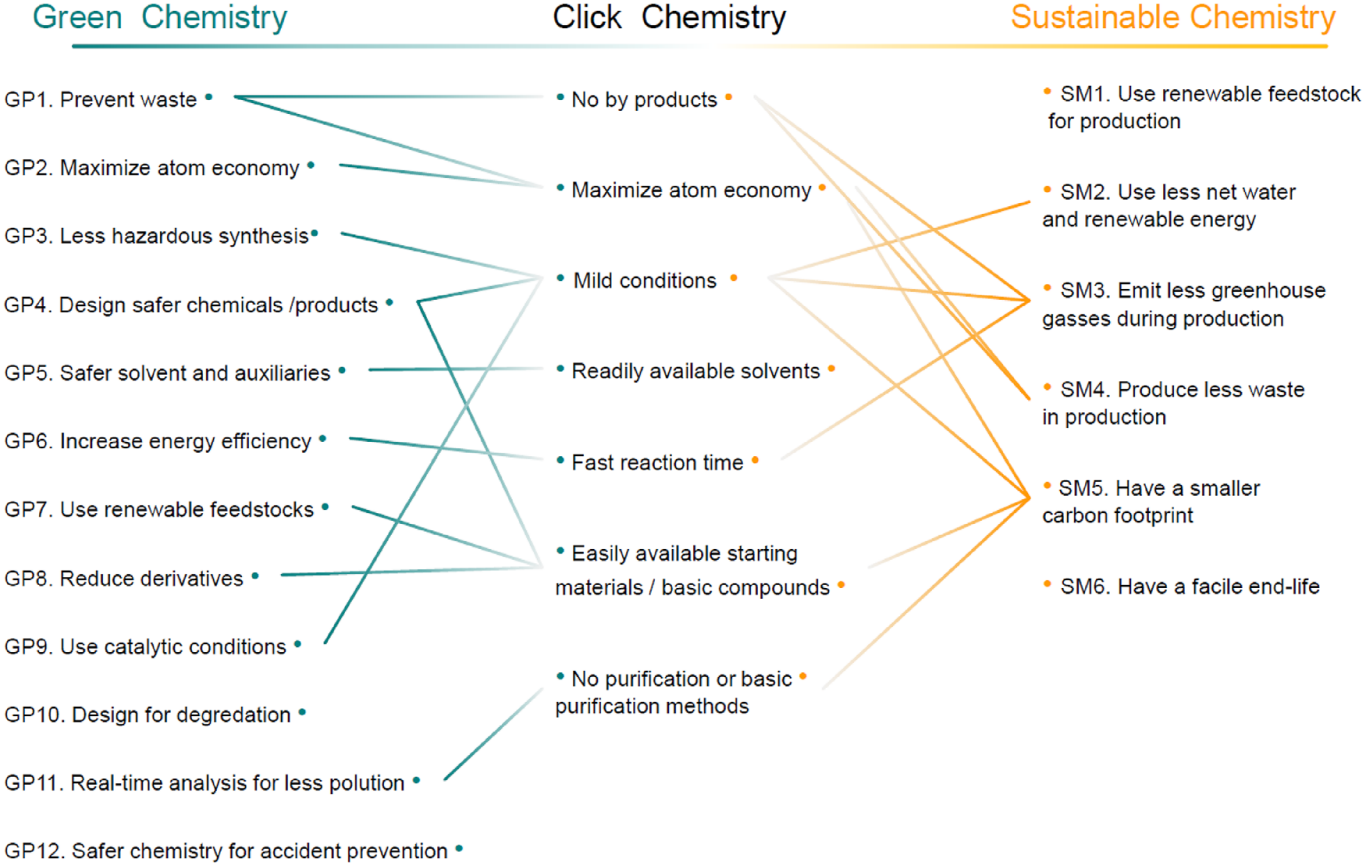
The principles of Green Chemistry principles (GP) and Sustainable Chemistry metrics (SM) and their common grounds with click chemistry.

If one were to evaluate how click chemistry fulfils the criteria of Green Chemistry, it becomes clear that its philosophy emphasizes the quick and reliable synthesis of substances by joining antagonist units together (Figure 4). In other words, click chemistry aligns well with Green Chemistry principles in several ways, exemplary:

- **Reducing Hazards**: Click chemistry typically utilizes simple, efficient reactions that generate minimal or no harmful by‐products, reducing the risks associated with traditional chemical synthesis.
- **Enhancing Efficiency**: The reactions in click chemistry often exhibit high yield and atom economy, meaning that the maximum number of atoms from the reactants are incorporated into the final product, minimizing waste.
- **Producing Harmless By‐products**: One of the core principles of Green Chemistry is to avoid harmful by‐products, and click chemistry often satisfies this by ensuring that the side products are benign or easily removed.

However, azide‐based click chemistry presents inherent drawbacks due to the energetic nature of azide derivatives. Although the use of high molar mass of azides reduces their explosive nature, it still involves hazardous materials and violates the primary principle of Green Chemistry, which is prevention. On a positive note, Zhang et al. reported the enzymatic synthesis of azide derivatives [16], still a significant part of producing azide derivatives are based on azidation with sodium azide, which is known its explosive nature.

## 3 Sustainable Chemistry

Linguistically, “sustainability” originates from the Latin “sustinere,” meaning to uphold or maintain, evolving through French “soutenir,” to support. It has evolved from its origins in forestry and ecology to become a cornerstone of modern development in the field of chemistry. The concept, popularized by the 1987 Brundtland Report by the United Nations, emphasizes meeting the importance of present needs without compromising the ability of future generations to meet their own. Generally, “*Sustainable Chemistry should utilize resources, including energy, at a rate that allows for natural replenishment, while ensuring that waste is generated more slowly than it can be naturally remediated”* [17]. To achieve this goal, Sustainable Chemistry aligns with the twelve principles of Green Chemistry, serving as a framework to emphasize that. Sustainable Chemistry prioritizes production processes that promote increased product value while aligning with the objectives of preserving and improving production standards regarding human life and the environment. The realization of sustainability in chemistry, particularly in polymer science, can be summarized through the below mentioned in six main Sustainable Chemistry matrices (SM):

SM1. Use renewable feedstocks for production.
SM2. Use less net water and nonrenewable energy in production.
SM3. Emit less greenhouse gases during production.
SM4. Produce less waste in production.
SM5. Have a smaller carbon footprint.
SM6. Have a facile end life.

By adhering to these pillars, Sustainable Chemistry aims to drive innovation, promote economic growth, and protect human health and the environment. While azide‐based click chemistry offers several sustainable attributes—efficiency, high selectivity, and low waste generation—the overall sustainability process is compromised by the inherent risks and challenges associated with azide reagents. To fully unlock the sustainability potential of azide‐based click chemistry, addressing these issues through improved reagent design, strict safety protocols, and advanced waste management is essential.

## 4 Alkyne‐Azide Cycloaddition (AAC)

The Huisgen dipolar cycloaddition between an azide and alkyne to afford a 1,2,3‐triazole has been highlighted as a powerful class of concerted click reaction [18, 19] and become the archetypal exemplar of the click chemistry philosophy [20]. Since then, a diverse array of azide‐alkyne cycloaddition click reactions have been employed in material science with different activation methods (Table 1).

**TABLE 1:** Overview of the main azide‐alkyne cycloaddition (AAC) reaction activation systems, highlighting reaction notes, sustainability aspects, and key references. R, R1 and R2 are representing aryl or aliphatic molecules. Green/Sustainable Chemistry scales justifications are given in detail in Table S1.

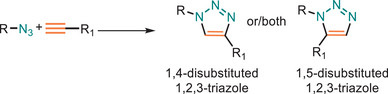					
Alkyne Reagent	Reaction Notes	Adduct(s)	Green/Sustainable Chemistry Scale^a^	Ref.	
				Review	Research
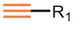	**TAAC**: Isomeric mixture, polymerization temperature up to 70°C, sourced from available bio‐based materials.	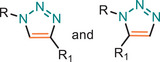	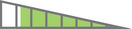	[21, 26, 33]	[34, 35, 36]
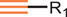	**CuAAC**: Potential for catalyst recycling, room temperature, accessible spatiotemporal control, partial control on isomer formation, compatible with microwave or photo‐induced systems, limited to terminal alkynes.	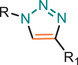	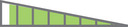	[37, 38, 39]	[5, 40, 41, 42, 43, 44, 45, 46, 47, 48, 49, 50]
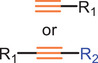	**MAAC**: Versatile transition metal catalyst options (Ru, Ag, Au, Ni, Zn) with potential for recycling, selective isomer formation depending on catalyst, ambient temperature, compatible with microwave or photo‐induced systems, possibility of utilizing internal alkynes.	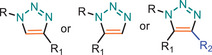	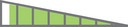	[51, 52, 53]	[54, 55, 56, 57, 58, 59, 60]
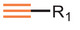	**MFAAC**: Metal‐free catalytic system, limited organocatalyts options, partial control over isomer formation.	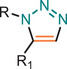	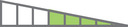	[61]	[62, 63]
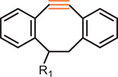	**DSPAAC**: Metal‐free process, available spatiotemporal control, limited and challenging monomer options.	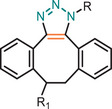	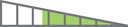	[64]	[65, 66]

TAAC: Thermal Activated AAC, CuAAC: Copper‐catalysed AAC, MAAC: Metal‐catalysed AAC, MFAAC: metal‐free AAC, DSPAAC: Strain‐promoted double AAC.

Although click reactions have predominantly been used for the post‐functionalization of preformed polymers in polymer science [21, 22], significant efforts are now being directed toward developing click chemistry as an efficient polymerization method—often referred to as click polymerization [23, 24, 25]. The click polymerization approach not only streamlines the synthesis of polymers with precisely controlled macromolecular architectures and functionalities but also expands the horizon for materials design by integrating diverse building blocks under mild reaction conditions while providing rapid and selective bond formation with high atom economy [26]. Crucially, the incorporation of the triazole ring into the structure of the materials grants several properties, as detailed below, that make the materials highly useful for a wide range of applications:

- **Thermal properties**: The triazole ring, particularly in poly(triazole)s contributes to high thermal stability with decomposition temperatures often exceeding 170–200°C. This makes densely triazole‐containing polymers well‐suited for use in high‐temperature required applications.
- **Chemical stability**: The triazole structure enhances the chemical stability. Their five‐membered ring structure, containing three nitrogen atoms, contributes to their robustness under hydrolytic, oxidative, and reductive conditions. This stability is further enhanced by their aromaticity and the strong dipole moment of the triazole ring, which provide resilience to harsh environments such as oxidative or corrosive [27].
- **Coordination chemistry**: The nitrogen atoms in the triazole ring are capable of coordinating with various metal ions, such as Cu, Ru, Pd, Ni, and Zn [28]. That can allow to design of different hybrid materials such as metal‐organic frameworks (MOFs) and as a ligand in catalysis. This property has been exploited in drug delivery and sensors [29].
- **Bioactivity**: The triazole ring is known for its bioactivity due to the tendency to engage with proteins, enzymes, and receptors within organisms, particularly in the context of medicinal chemistry. It is found in a number of antifungal, anticancer, antiviral, and antibacterial agents [30].
- **Electronic properties**: The triazole ring can delocalize electrons, which can enhance the conductivity and electronic properties of materials [31]. Triazole‐based materials are therefore of significant interest for applications in organic electronics and semiconductor technologies.
- **Polarity and hydrogen bonding**: The triazole ring contains two pyridine‐like nitrogen atoms with lone pairs of electrons, which function as hydrogen bond acceptors, analogous to the role played by the oxygen atom in amides [32].

Following a concise overview of the key features of activation methods, the AAC reaction will be illustrated with additional green and sustainable examples. This guideline is designed to assist researchers in aligning their expectations for green and sustainable azide‐based click chemistry with state‐of‐the‐art practices, enabling them to select the most appropriate activation methods for their study. Ultimately, our objectives are to facilitate their contribution to the development of next‐generation polymeric materials derived from click polymerization methods. Within this work, we also provide a comprehensive summary of click polymerization principles from a sustainability‐focused perspective, highlighting potential areas for integration of greener practices into existing methodologies (Table 2).

**TABLE 2:** Overview of metal‐catalysed azide‐alkyne cycloaddition (AAC) reaction with click polymerization references.

Metal Catalyst	Notes	Adducts	Ref.^a^ Research
RuAAC	Effective catalysis, regioselective synthesis, costly catalyst design, recyclability challenges	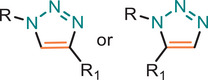	[140]
NiAAC	Effective catalysis, regioselective synthesis, limited availability to the Nickel in the future, recyclable catalyst	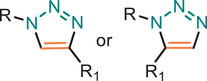	[54]
AgAAC	Effective catalysis, regioselective synthesis, affordable and cost‐effective catalyst	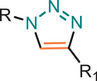	—
RhAAC	Effective catalysts, monomer diversity, rare and costly metal utilization in the catalyst design	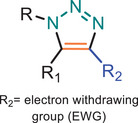	[141]
ZnAAC	Effective catalysis, recyclable catalyst, lower toxicity	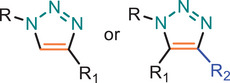	—
IrAAC	Effective catalysts, monomer diversity, costly catalyst design, high toxicty	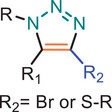	[55]

^a^ Click polymerization references are given in the Ref. column.

### 4.1 Evaluating AAC Polymerization Under Green and Sustainable Chemistry Criteria

Herein, in Table 2, we evaluate the AAC reactions together with the reaction notes and resulting triazole adducts. The Green/Sustainability scale has been developed based on the criteria outlined in Table S1, which summarizes the features of click chemistry, specifically the azide‐alkyne cycloaddition (AAC) reaction, and maps them against the 12 principles of Green Chemistry as well as the 6 matrices of Sustainable Chemistry. Detailed scoring of individual AAC reactions can also be found in Table S1. Furthermore, references have been divided into two subgroups as review and research articles, which aimed to enable readers to easily find references.

The core principles of click chemistry, particularly for azide‐alkyne cycloaddition reactions, have been integrated with the pillars of Green and Sustainable polymer chemistry. The key features of click reactions have been categorized into three main groups: catalytic systems, reaction conditions, and resulting materials, all of which align significantly with Sustainable Chemistry objectives. Both catalytic systems and reaction conditions share sustainability considerations, such as utilizing renewable feedstocks, minimizing net water consumption and non‐renewable energy usage, reducing greenhouse gas emissions, generating less waste, and achieving a lower carbon footprint. Additionally, the review emphasizes the synthesis of functional polymeric materials with a focus on their recyclability and ease of end‐of‐life management, directly corresponding with sustainability criteria. While all click polymerization reactions inherently showcase high chemical efficiency and practicality, the presented sustainability scale specifically highlights areas for improvement while increasing/decreasing green colour on scale bar, aiming to pinpoint even greener and more sustainable chemistry practices.

### 4.2 Thermal Activated Azide‐Alkyne Cycloaddition (TAAC)

The thermal activated azide‐alkyne cycloaddition click polymerization, utilizing the step‐growth mechanism between antagonist azide and alkyne‐containing monomers, remains one of the earliest and most straightforward method for synthesizing poly(triazoles) (PTAs). Designing monomers for this process requires a careful balance of several factors, including functional group reactivity, spacer length, electronic properties, and safety considerations. This optimization is essential to achieve controlled polymerization, which in turn ensures the formation of polymers with tailored thermal stability, mechanical properties and specific functional characteristics. In particular, the design of AB‐type monomers is fundamental for achieving high‐performance PTA materials, which find broad applications across diverse fields such as coatings, adhesives, biomedical devices, and optical materials.

**FIGURE 5:**
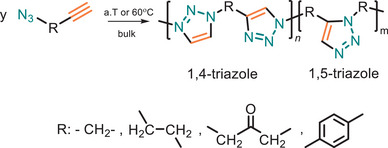
Synthesis of polytriazole (PTA) from AB‐type monomer in bulk condition.

The pioneering AB‐type monomer for click polymerization was reported in 1966 by Baldwin and Parker [34, 35], following 1,3‐dipolar cycloaddition reaction between alkyne and azide to yield a brittle dark yellowish‐brown polymer (Figure 5). The polymerization reactions were performed under bulk conditions at either ambient temperature or 60°C depending on the specific structure of monomer. The resulting PTAs exhibited high decomposition temperatures (170 to 200°C). From a sustainability perspective, this polymerization method is particularly valuable due to its efficiency, selectivity, and ability to produce functional polymers with tailored properties. However, challenges remained, especially regarding the hazardous nature of azide monomers, which exhibit high sensitivity and can be dangerously explosive, with impact sensitivity higher than nitroglycerin. This safety concern has prompted researchers to explore alternatives that ensure both Green Chemistry principles and safe working conditions while maintaining the effectiveness of the polymerization.

Hyperbranched polymers (HBPs) are important class of branched macromolecules, often synthesized with a high density of functional groups. Their synthesis can be highly efficient, particularly when utilizing AB_2_‐type monomers, which contribute to favorable atom economy [67, 68, 69]. In pioneering work from Voit and her team proposed the formation of hyperbranched polymer from AB_2_‐type monomer containing 1,2‐disubstituted alkynes. Upon low‐temperature (up to 60°C) polymerization, fully soluble products of high molecular weight (with *M*_n_ values of 10 000 g·mol^−1^) could be obtained with a degree of branching of 50%.

The resulting products bear a large number of alkyne or azide groups which can allow further end‐group modifications or crosslinking formation, enabling the development of self‐healing materials while extending the lifespan of products [36].

Indeed, numerous further efforts over the past decades have focused on TAAC polymerization, building upon foundational studies to develop innovative synthetic methodologies [70, 71, 72, 73, 74, 75, 76, 77, 78]. While these approaches demonstrate significant potential, they predominantly center on advancing synthetic techniques rather than addressing sustainability concerns. Consequently, these approaches will not be covered in this review in detail.

The traditional thermal Huisgen 1,3‐dipolar cycloaddition between azides and alkynes yields a mixture of 1,4‐ and 1,5‐disubstituted 1,2,3‐triazole isomers, lacking regioselectivity. This non‐specificity can complicate structure and properties of the polymer. However, Tong and co‐workers have further investigated systematically TAAC reactions while using various azide and alkyne monomers [79]. The key innovation in their work lies in the use of electron‐withdrawing carbonyl groups in the diyne monomers, which significantly enhance the reactivity and regioselectivity of the reaction, yielding 1,4‐disubstituted triazole units with remarkable precision (up to 95% regioselectivity). This advancement addresses the primary drawbacks of previous thermally induced polymerizations, which suffered from low efficiency, slow reaction rates, and regio‐random product distributions.

Despite the potential hazards of azides, advances in TAAC click polymerization have significantly enhanced sustainability and environmental compatibility. In 2009, Drockenmüller and colleagues developed a starch‐derived AB‐type monomer as a potential alternative to petrochemical‐based AB monomers [80]. Their polymerization protocol, performed in bulk, minimized the carbon footprint by utilizing renewable feedstocks. However, the reaction required temperatures exceeding 200°C in order to obtain quantitative conversion of the monomer and obtaining high degree of polymerization, which contradicts the principles of Design for Energy Efficiency, highlighting a trade‐off between sustainability and reaction conditions.

In line with the growing emphasis on sustainable development, new building blocks from castor, canola, corn, soybean and linseed oils were also used in this thermal‐driven click polymerization reaction [81]. Notably, the polymerization temperature (100°C) was significantly lower than previous reports, enhancing energy efficiency. However, the utilization of these edible oil derivatives raises concerns regarding food and feed competition, which contradicts key sustainability principles. To address this, researchers are increasingly exploring non‐food‐based alternatives, such as algae‐derived oils, lignocellulosic biomass, and industrial waste lipids, to ensure a more sustainable and environmentally responsible approach to polymer synthesis. In fact, Narine and colleagues have utilized lipid‐derived, readily available, and cost‐effective feedstocks as monomers for PTA synthesis via AA‐BB step‐growth polymerization method [82]. This approach adheres to Green and Sustainable Chemistry principles by the possibility of in situ polymerization and modification for biocompatibility and tunable functionalities offers a sustainable method for polymer (up)cycling, reducing waste and enhancing environmental compatibility compared to traditional methods. Still, the reliance on an AA‐BB monomer system may introduce challenges such as the need for precise stoichiometric balance, potentially leading to unreacted monomers and increased waste.

**FIGURE 6:**
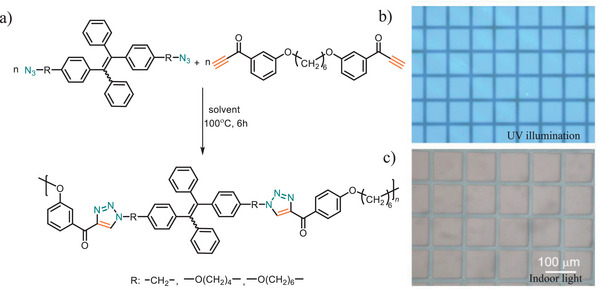
(a) Synthesis of PTAs by metal‐free and solvent‐free thermal click polymerization. (b) 2D fluorescence image of PTA polymer (R: ‐CH_2_‐) under UV irradiation. (c) 3D photo pattern generated by UV irradiation at indoor light. Adapted with permission from ref [79]. Copyright 2025, Wiley‐VCH GmbH.

Furthermore, the incorporation of aggregation‐induced emission active units into PTAs enhances optical applications and enables self‐reporting for real‐time monitoring, offering a sustainable, eco‐friendly solution aligned with Green Chemistry principles. By simply heating for 6 h, two azide‐functionalized tetraphenyl ethene and bis(aroyl acetylene) monomers undergo polymerization, yielding high‐molecular‐weight poly(aroyl triazole)s with a weight average molecular weight of *M*_w_ = 16 300 g·mol^−1^ (85% yield, Figure 6). The obtained polymers are completely soluble in common organic solvents and exhibit thermal stability up to 350°C. However, the monomers used were primarily derived from petroleum‐based azide compounds, raising sustainability concerns due to their environmental impact. To enhance the sustainability of these polymers, future research should be dedicated on exploring monomers from renewable resources and/or greener synthetic approaches. Further study showed that the obtained polymer has excellent film‐forming ability and self‐healing properties, attributed to the presence of unreacted azide and acetylene moieties [83]. This feature highlights the potential for facile end‐of‐life management, enhancing the sustainability of the materials.

Looking to the future, there is considerable promise in optimizing the monomer design, exploring non‐food renewable feedstocks, and lowering reaction temperatures to further improve energy efficiency. Additionally, advancing solvent‐free approaches and developing scalable techniques will be key to enabling the industrial‐scale adoption of this method. As these challenges are addressed, TAAC click polymerization will continue to evolve as a valuable tool for sustainable polymer synthesis and green chemistry.

### 4.3 Copper‐Catalyzed Azide‐Alkyne Cycloaddition (CuAAC)

In sustainable and Green Chemistry, catalysts play a crucial role in improving efficiency, reducing waste, and minimizing environmental impact [84]. Indeed, the choice of catalyst directly impacts energy consumption, waste generation, and feedstock utilization.

Subsequently, the discovery of the copper(I)‐catalyzed alkyne‐azide cycloaddition (CuAAC) reaction, which proceeds regio selectively at a fast rate under mild conditions, has greatly enhanced the synthetic value of this transformation [85, 86]. The absence of side products and the relative insensitivity of reactions to the concentrations of alkynes and azides have made it widely applicable, even at very low functional group concentrations. In the reaction, Cu(I) can be obtained directly from Cu(I) salts or complexes, but its thermodynamic instability, makes it prone to oxidation and disproportionation, leading to side‐products in organic solvents. To mitigate these issues, Cu(I) is often generated in situ via Cu(II) reduction with ascorbate or through comproportionating of Cu(II) and Cu(0). Stabilizing ligands, such as 1,2,3‐triazoles, are also used to prevent oxidation and maintain catalytic activity [87, 88, 89, 90]. Shortly after the development of the CuAAC reaction, polymer chemists began to explore the possibility of utilizing this click reaction for the synthesis of dendritic oligomers and hyperbranched polymers [5, 36]. In 2004, Voit et al. attempted to synthesize hyperbranched poly(triazole)s (hb‐PTAs) using copper (II) sulphate (CuSO_4_) and sodium ascorbate to polymerize 3,5‐bis(propargyloxy)benzyl azide [36]. However, the reaction yielded an insoluble rubbery material, and the AB_2_ monomer used was challenging to prepare and prone to self‐oligomerization during storage. Despite these challenges, this marked the first attempt at CuAAC click polymerization. Since then, the CuAAC has been widely used to control the macromolecular architecture enabling the synthesis of hyperbranched polymer and crosslinked polymer [22].

**FIGURE 7:**
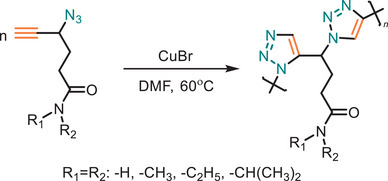
CuAAC polymerization of 4‐Azido‐5‐hexynamide derivatives to yield thermoresponsive polymers, which can function as self‐healing materials, effectively extending product lifespan, reducing waste, and promoting sustainability.

Beyond synthetic methodology, designing materials with a facile end‐life strategy is crucial for sustainability, ensuring efficient degradation, recycling or repurposing to minimize environmental impact. Thermoresponsive polymers, which can autonomously repair damage in response to temperature changes, function as self‐healing materials, extending the lifespan of product and reducing waste. The resulting polymer from CuAAC polymerization features a highly polar backbone that is densely populated with 1,4‐disubstituted 1,2,3‐triazole rings [50, 91, 92, 93, 94, 95]. This structural feature influences chemical and physical properties, often resulting in poor solubility in conventional organic solvents. To address this Winnik and his team have designed thermoresponsive water‐soluble polymers via CuAAC polymerization [49]. 4‐azido‐5‐hexynamide monomers which were designed, which polymerized under ambient conditions within 1 h, forming thermoresponsive polymers with a densely packed 1,2,3‐triazole backbone (Figure 7). Post‐polymerization grafting of hydrophilic side chains via nucleophilic substitution allowed precise tuning of the lower critical solution temperature, enhancing their thermoresponsive behavior.

**FIGURE 8:**
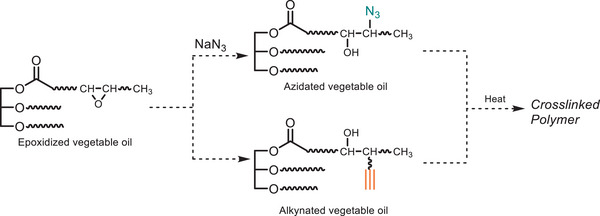
Thermal assisted polymerization of vegetable oil‐derived monomers via AAC reaction enables using renewable feedstocks.

The ease of synthesizing azide derivatives via nucleophilic substitution using sodium azide or trimethylsilyl azide from readily available reagents, such as alkyl halides, tosylates, or mesylates, under mild conditions facilitates the incorporation of bio‐based sources into azide‐alkyne cycloaddition reactions [96]. Accordingly, Hong et al. derivatized the fatty acid chains with azide and alkyne structure through a ring‐opening nucleophilic addition reaction [97]. Microwave‐assisted synthesis is a significant advancement in chemical synthesis, aligning with objectives of green chemistry. By using microwave irradiation for heating reactions, not only reaction efficiency improved but also reaction time is reduced‐along minimized resource consumption compare to conventional heating methods [98]. They tested solvent and catalyst‐free conventional thermal CuAAC reactions of the alkynated and azidated soybean oil derivatives (Figure 8) [97]. The solvent‐free thermal polyaddition process (heating at 100°C) outperformed the traditional ambient temperature CuAAC polyaddition by producing higher yields (96.4%) in shorter reaction time (12–24 h compared with 48–72 h required for the CuAAC polymerization). From a sustainability perspective, this process is superior to CuAAC by offering reduced environmental footprint, cleaner (i.e., residual catalyst‐free) product formation, and simplified operation. However, to maximize its green potential, further efforts are needed to source renewable feedstocks and optimize energy use in thermal polymerization.

**FIGURE 9:**
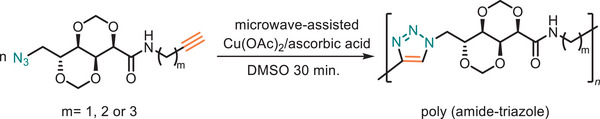
Microwave‐assisted polymerization of AB‐type monomers via the CuAAC reaction enables rapid and efficient synthesis, yielding high‐molecular‐weight polymers with enhanced thermal properties.

Appukkuttan et al. have developed a microwave‐enhanced, fast, and efficient three‐component reaction for the generation of 1,4‐disubstituted1,2,3‐triazoles in a complete regioselectivity [99]. Cardoso‐Ortiz et al. further showed that microwave irradiation can be used CuAAC reaction for synthesizing quinolone derivatives under mild conditions (125W_max_, 140°C and 10 min) resulting in good yields (80%) [100]. Djik et al., have demonstrated that the microwave‐assisted CuAAC reaction can be successfully employed in the synthesis of degradable peptide based polymers containing unprotected functional amino groups [101, 102]. These polymers were degradable by chymotrypsin and trypsin proteases, making microwave‐assisted AAC polymerization a convenient method for producing biomedical polymers. Fidalgo and coworkers also showed that biobased α‐azide‐ω‐alkyne monomers can be polymerized by microwave‐assisted CuAAC reaction yielding poly(amide‐triazole)s [103]. They designed three different AB‐type self‐reacting monomers from D‐glucono‐1,5‐lactone with a straightforward route from 2,4;3,5‐di‐O‐methylidene‐d‐gluconic acid (Figure 9). Through microwave‐assisted CuAAC click polymerization, polymers with high number average molecular weights values (*M*_n_ = 17 000 g·mol^−1^, 30 min.). were obtained in just 30 min. These poly(amide‐triazole)s also exhibited higher glass transition temperature (*T*_g_, i.e.>180°C)_1_ with longer methylene groups in the repeating units.

**FIGURE 10:**
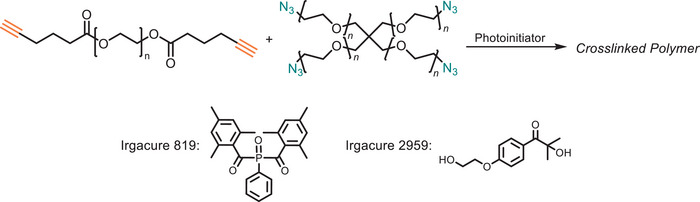
A light‐triggered CuAAC reaction where a photoinitiator reduces Cu(II) to Cu(I) under UV or visible light irradiation, enabling controlled and efficient click polymerization with spatial and temporal precision.

The use of copper in the reaction lowers the reaction temperature to 25°C and introduces a new avenue for light‐induced CuAAC reactions, enabling spatial (specific location) and temporal (specific times) control over‐click polymerization [104]. Light‐induced click reactions merge of photochemistry and click chemistry principles, offering precise and efficient polymer synthesis. By harnessing light as a trigger, these reactions offer exceptional temporal and spatial control over the polymerization process, allowing researchers to tailor material properties with high precision [105, 106, 107, 108, 109]. Bowman and coworkers pioneered a photoinitiated Cu (II) reduction using a photoinitiator under visible light (10 mw cm^−2^, 400–500 nm) irradiation (Figure 10). UV irradiation of an aqueous solution of azide and alkyne species in the presence of Type I initiator α‐cleavage‐type photoinitiator (Irgacure 819, Irgacure 2959) and copper sulfate pentahydrate results in the generation of radicals that subsequently reduce Cu (II) to Cu (I).

Yagci et al. further developed a mild and highly versatile visible light‐induced CuAAC click reaction via photoinduced electron transfer using free radical photo initiators from near UV region to the visible light spectrum (250–700 nm) [110]. Various initiators were tested for their efficiency in Cu (II) reduction during CuAAC‐mediated small molecule synthesis, revealing that Type I initiators were the most effective. The transient excited states (singlet and triplet) of type I initiators have very short lifetimes, preventing quenching by metal ions. In contrast, Type II initiators showed lower efficiency due to the bimolecular reaction of reactive radicals, which have relatively lower quantum yields. Among the tested initiators, a titanocene‐based photoinitiator exhibited the highest efficiency for CuAAC click reactions. This methodology has been used successfully applied for the functionalization of polymers, paving the way for the development of a new light‐induced system for CuAAC [111, 112, 113, 114]. However, many organic initiators are derived from petroleum‐based sources and exhibit toxic and hazardous properties. Additionally, residual organic initiators or their byproducts may require additional purification to avoid contaminations, conflicting Sustainable and Click Chemistry requirements.

The use of heterogeneous catalysis in organic synthesis is becoming increasingly important from a Green Chemistry perspective as it facilitates catalyst recovery and reuse, aligning well with click chemistry principles. Copper species can act as catalysts either in a homogeneous form, dispersed uniformly within the reaction mixture, or in a heterogeneous form, immobilized on a host material. Various copper‐based heterogeneous catalytic systems have been developed using different support materials, including organic frameworks, carbon materials, amorphous inorganic solids, and structured inorganic solids [115]. Additionally, copper can be employed in diverse forms, such as nanoparticles or nanoporous copper. Pale and coworkers have reviewed the heterogeneous CuAAC click reaction in detail, and emphasize the growing relevance of heterogeneous CuAAC as a sustainable and practical alternative for various applications [116].

**FIGURE 11:**
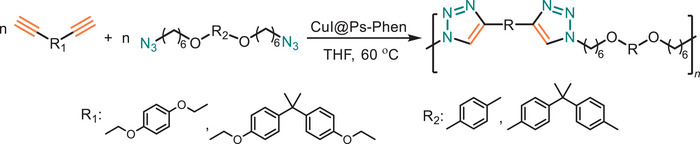
Polymer supported copper‐based heterogeneous catalysts enable efficient, recyclable, and greener CuAAC reactions, minimizing metal contamination and waste.

As previously mentioned, Cu species act pivotal role for catalyzing the triazole formation. Therefore, one of the most important parameters is to maintain the Cu(I) concentration at a sufficient level during the reaction. The use of Cu(II) source with addition of a reducing agent in large excess is often preferred [87, 117]. Tang and coworkers have integrated copper into the polymeric catalyst based on phenanthroline by modifying Merrifield resin (crosslinked polystyrene that carries a chloromethyl functional group) at a ppm level (Figure 11) [118]. It is known that phenanthroline can form a stable complex with Cu(I), which has been used as a supported catalyst. In this transformation, the supported catalyst has promoted the synthesis of functional PTA in tetrahydrofuran (aka THF). Under these reaction conditions, catalysts can be used up to 4 times, and moreover, residual Cu(I) can be lower by around 25 times compared to the conventional Cu(I) catalyst usage. However, the synthesis of polymeric catalysts predominantly relies on petroleum‐derived monomers, and the reusability of these catalysts remains inadequately explored.

Wu et al. have further showed enhanced catalyst recyclability of support material by using Amberlyst A‐21 resin for Cu catalyst. In fact, by employing copper species at ppm levels to, they effectively catalysed click polymerization, achieving high molecular weight polymers, even after the fourth recycling of the catalyst [119].

**FIGURE 12:**
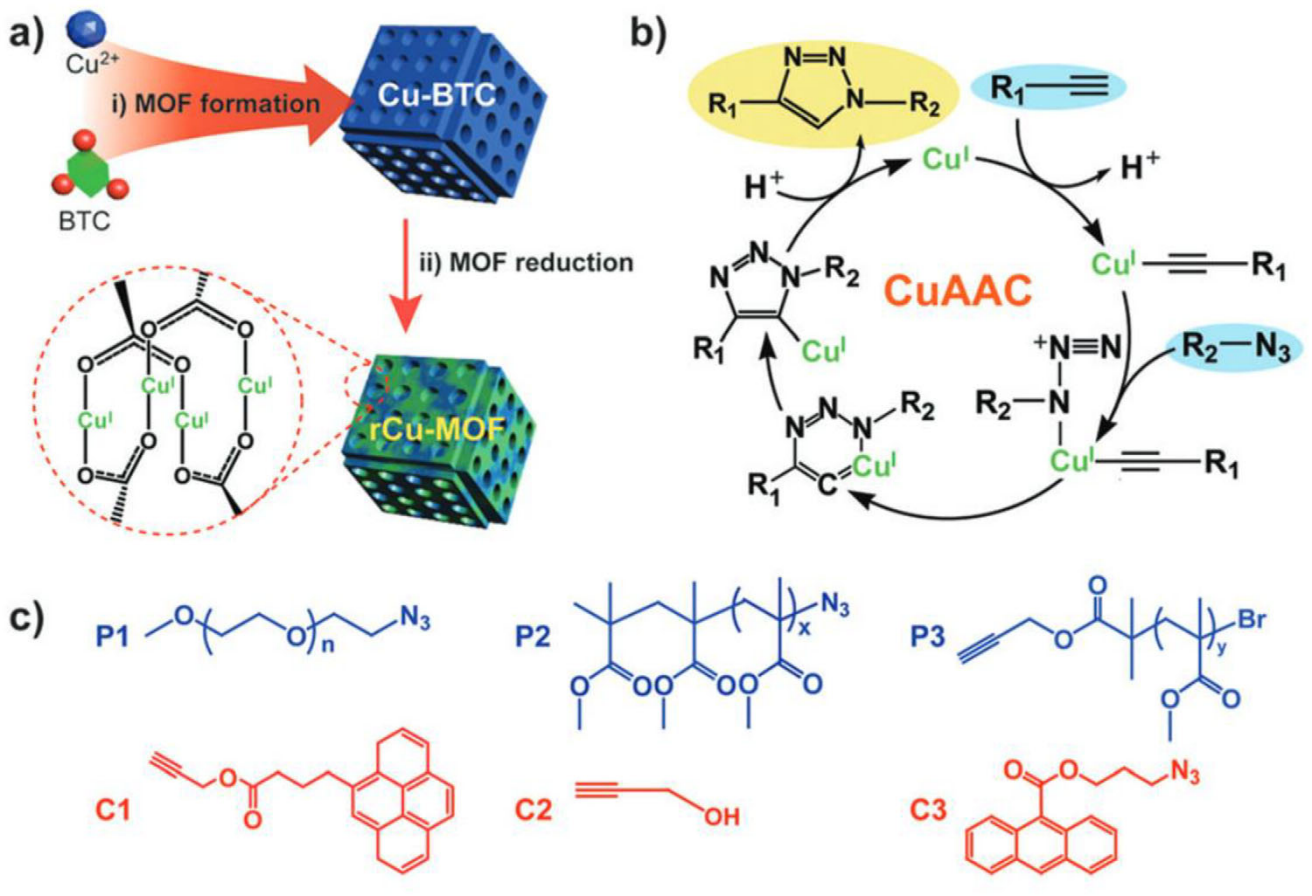
(a) Schematic illustration of the preparation of a reduced copper metal–organic framework (rCu‐MOF): (i) coordination reaction between Cu2+ and benzene 1,3,5‐tricarboxylate (BTC) and (ii) reduction of the Cu‐BTC in the presence of hydroquinone at 150°C. (b) Schematic representations for various polymer chain end functionalizations and coupling reactions utilizing rCu‐MOFs via CuAAC chemistry. (c) Chemical structures of the synthesized precursors for the CuAAC reactions. Reproduced (Adapted) with permission [120]. Copyright 2025, Royal Society of Chemistry.

Copper can be integrated not only into organic materials but also into COFs (covalent organic frameworks), MOFs [120, 121, 122, 123, 124] (metal‐organic frameworks), and polymeric networks [125]. Corma and co‐workers were the first to demonstrate that a Cu‐MOF structure could efficiently catalyse 1,3‐dipolar cycloaddition reactions, achieving high yields [126]. They also showed that a solvent‐free approach, in which pre‐treating the catalyst with excess alkyne enhanced efficiency, allowing easy catalyst recovery and alkyne recycling. The catalyst maintained its activity and selectivity for at least six consecutive times. The first example of reduced Cu‐MOF (rCu‐MOF) catalytic properties also has been reported by Qiao and coworkers in azide‐alkyne click reaction for polymer chain end modification and block copolymer formation [120]. Indeed they employed green synthesis of MOFs based on d 1,3,5‐benzene tricarboxylic acid under relatively mild conditions (such as reducing MOF structure at 150°C), producing less waste than conventional procedures (Figure 12). These properties, combined with its ease of synthesis, lack of heavy metal contamination and high structural stability, make the rCu‐MOF an attractive catalytic material for industrial applications. Nevertheless, while MOF structures have been used in click ligation reactions, they have not been involved in click polymerization reactions yet.

Vigroux and coworkers further showed that the controlled release of Cu(II) species from a mesoporous MOF structureinto a reducing aqueous medium effectively promotes the click reaction [122]. The first MOF system was synthesized using 2‐amino terephthalic acid, which was impregnated with copper sulfate. This approach mitigates substrate degradation by preventing local concentrations of reactive Cu species in the reaction media. The resulting material, Cu@MOF1, demonstrated sustained copper release in polar aqueous solvents containing sodium ascorbate, making it a practical heterogeneous Cu(II) source for CuAAC. At a loading level of 7.5 mg copper sulfate per 10 mg of MOF1 (27% of capacity), Cu@MOF1 maintained excellent catalytic efficiency for up to five cycles.

**FIGURE 13:**
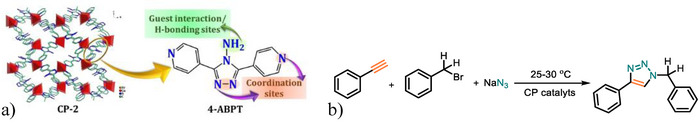
(a) Demonstration of Cu(I)‐CP catalysts with active sides. (b) Azide‐alkyne cyclodaddition reactions via heterogenous Cu(I) catalyst under ambinet consitions. Reproduced (Adapted) with permission [129]. Copyright 2025, Wiley‐VCH GmbH.

Coordination polymers (CPs) can often be regenerated and reused, particularly in catalytic or adsorption processes. CPs were also investigated as a potential catalyst host for Cu(I) [125, 127, 128]. Puzari and coworkers reported 2‐picolinic acid‐based Cu(II) coordination polymers for CuAAC reaction. The 1D coordination polymer was synthesized via a solvothermal method, exhibited high catalytic activity in the selective synthesis of 1,4‐disubstituted 1,2,3‐triazoles achieving excellent yields (>95%) under mild conditions (aqueous ethanol, 70°C). A mechanistic study confirmed that a Cu(I) catalytic cycle facilitated by in situ reduction of Cu(II) by sodium ascorbate. The heterogeneous nature of the catalyst enabled an easy recovery and reuse over multiple cycles without significant loss of activity [128]. A recent study from the Sarma group has shown Cu(I) coordination polymers featuring both Lewis acid and basic sides (Figure 13) [129]. The respective functional groups facilitate the proximity of analytes and substrates to the metal centre, enhancing catalytic efficiency. The catalyst enabled the regioselective formation of 1,4‐disubstituted 1,2,3‐triazoles with excellent yields (>95%) across a broad range of substrates. Its heterogeneous nature facilitated easy recovery and reuse for multiple reaction cycles without significant loss in catalytic performance. The mild reaction conditions, combined with the absence of external ligands and environmentally benign media further highlights the alignment of the polymer with green chemistry principles.

Yagci and his team systematically worked on developing new photocatalytic systems for azide alkyne click reactions [130, 131, 132] One of the works from his group enabled using Cu(I) coordination polymer based on benzophenone 4,4′‐dicarboxylate ligand. The zig‐zag 2D structure of catalyts provided an abundance of active Cu(I) sites which effectively catalyzed the CuAAC reactions at ambient temperature. The catalyst was easily recovered and reused up to 5 times without significant loss of efficiency, emphasizing its sustainability [133].

**FIGURE 14:**
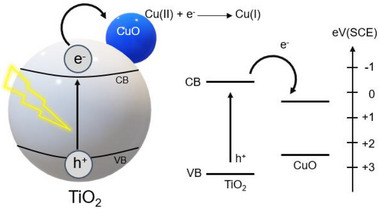
Proposed mechanism of electron transfer from the excited semiconductor to CuO nanoparticles to form catalytic Cu(I). Reproduced with permission from Ref. [134] Copyright 2014, American Chemical Society.

The heterogeneous photoactivated catalysts such as copper‐doped semiconductors have also been employed to facilitate azide‐alkyne click reactions [134, 135, 136]. Upon ligh exposure, these materials generate electrons that can transfer into nearby Cu nanoparticles, reducing the Cu(II) to Cu(I), which is the active species that catalyzed the reaction (Figure 14). This heterogeneous catalyst system is highly tolerant to air and moisture, addressing a common limitation of traditional Cu(I) catalysts, which often degrade or lose activity under such conditions. These catalysts can also be easily recovered and reused, enhancing both sustainability and cost‐efficiency. This study highlights the practical utility of Cu(I) coordination polymers for sustainable, high‐efficiency click chemistry applications.

Another recent study by the group of Sarma have introduced that a novel photocatalyst consisting of ultralow‐loading copper sulfide nanosheets supported on graphitic carbon nitride (g‐C_3_N_4_) that operates efficiently under visible light [137]. This catalyst harnesses the synergistic interaction between copper sulfide and g‐C_3_N_4_ to promote effective charge separation, leading to enhanced photocatalytic activity. Under visible‐light irradiation, the proposed catalyst (Cu_1.8_S‐GCN) enables the regioselective synthesis of 1,4‐disubstituted 1,2,3‐triazoles, a class of compounds commonly prepared through click chemistry with a high yield (∼99%). Notably, the use of ultralow amounts of copper sulfide not only minimizes the metal usage but also contributes to a more sustainable and environmentally friendly alternative to traditional methods that often require higher metal loadings or harsher conditions.

Even though proposed heterogeneous photocatalytic systems have been used in small molecule synthesis, they have not been employed in polymerization reactions. Indeed, their implementation in polmyerization can pave the way to a more sustainable perspective due to the recyclable nature of catalysts. The recyclability of catalyst is a critical factor in ensuring the sustainability of catalytic processes. To maximize the heterogeneous catalyst sustainability, following parameters should be fulfilled:

- **Robust catalyst supports**: Developing durable support materials (preferably from renewable feedstocks) that can withstand multiple reaction cycles without degradation, ensuring long‐term stability and activity.
- **Efficient recovery**: Development of straightforward and cost‐effective separation techniques, such as filtration, adsorption, magnetic recovery, or membrane‐based separations [138].
- **Catalyst regeneration**: Designing green and energy efficient regeneration processes to restore catalytic activity after deactivation caused by impurities or structural changes [139].
- **Reaction conditions**: Eliminating harsh reaction conditions to minimize catalyst wear and promote recyclability.

### 4.4 Metal Catalysed Azide‐Alkyne Cycloaddition (MAAC)

**FIGURE 15:**
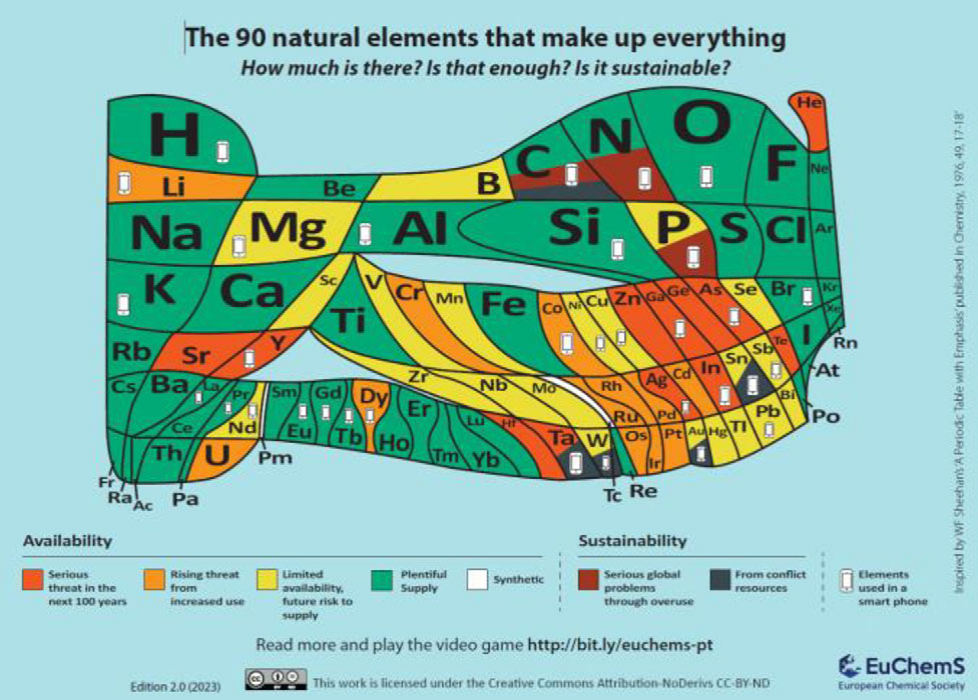
The 2024 EuChemS periodic table depicts element sustainability and causes of concern for future availability, including increased use and production from conflict resources, and impact on Earth system processes. EuChemS, reproduced from https://www.euchems.eu/euchems‐periodic‐table/ under a Creative Commons license CC BY‐ND.

To assess the choice of metal from a sustainability perspective, it is essential to consider the abundance of elements as represented in the EuChemS Periodic Table, which highlights both availability and extraction challenges (Figure 15). Transition metals such as Ru, Rh, and Pd exhibit exceptional catalytic activity, making them indispensable in various industrial and pharmaceutical processes. However, their increased use over the last two decades has raised significant concerns due to their scarcity, rising costs, and the environmental impact associated with their extraction and limited recyclability. Copper catalyst demonstrates high efficiency but has certain limitations including purification challenges, toxic metal accumulation, restriction to the formation of 1,4‐disubstituted triazoles, confinement to terminal alkynes, and potential future supply risks. Systematically, many studies have shown the AAC reaction can be realized by using different transition metal atoms such as Ru, Rh, Zn, Ag, and Ni, offering alternative pathways for catalyst development which some of the metal catalysts are mentioned in Table 1.

The 1,5‐disubstituted triazole rings are of greater interest due to the adjacent positioning of their substituents, which enhances their potential for cyclization reactions. Furthermore, their rigid cyclic structures make them attractive for materials science applications. In this regard, the Ru(II)‐catalyzed azide alkyne cycloaddition (RuAAC) reaction enables the selective formation of 1,5‐disubstituted triazole. Unlike CuAAC, this reaction is effective with both terminal and internal alkynes, thereby expanding the scope of alkyne reactants and advancing the field of click chemistry [142, 143]. Furthermore, based on Ru(II) catalyst structure both 1,4 and 1,5‐disubstituted 1,2,3‐triaole ring can be formed via RuAAC. The regioselective Ru(II) catalyst list are given in Table 3.

**TABLE 3:** Overview of RuAAC reaction and regioselective catalyst for 1,4‐ and 1,5‐disubstituted 1,2,3‐triazole ring.

Catalyst	Ref.^a^	Adduct	Ref.^a^	Catalyst
Ru(OAc)_2_(PPh_3_)_2_	[144]	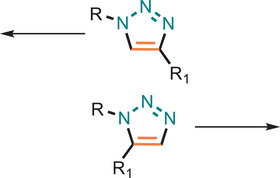	[142, 145, 146, 147, 148]	Cp*RuCl(PPh_3_)_2_
RuCl_2_(PPh_3_)_3_	[149]		[144, 147]	Cp*RuCl(COD)
RuHCl(CO)(PPh_3_)_3_	[150]		[144]	Cp*RuCl(NBD)
RuCl_2_(PPh3)_3_	[149]		[151, 152, 153]	[Cp*RuCl_2_]_4_
RuH_2_(CO)(PPh_3_)_3_	[154]			

^a^ Small molecule RuAAC click reaction references are given in the Ref. column.

**FIGURE 16:**
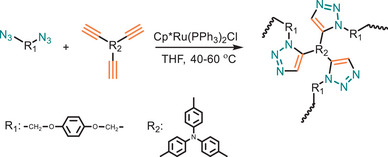
Syntheses of regioregular *hb*‐PTAs by RuAAC of diazides and triyne monomer.

Tang and his team have demonstrated that Cp*Ru(PPh_3_)_2_Cl and [Cp*RuCl_2_]_n_ catalysts effectively catalysed the click polymerization to synthesize high molecular weight 1,5 disubstituted hyperbranched polymers (Figure 16) [153]. By employing an A_2_ + B_3_ monomer design, they successfully prevented self‐oligomerization, yielding soluble polymers with enhanced properties compared to copper‐catalysed alternatives. These Ru‐based catalysts were found to exhibit high catalytic activity toward electron‐rich triyne monomers, enabling the click polymerizations to be a regioselective one, furnishing hyperbranched polymer (*hb*‐PTAs9 in high yields (>83%).

**FIGURE 17:**
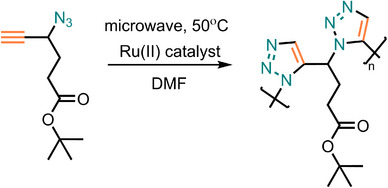
Microwave‐assisted *t*BuAH AB‐type monomer polymerization via Ru (II) catalyst.

Hashidzume and coworkers further explored click polymerization using two Ru(II) catalysts (Figure 17) [147]. They demonstrated that both Cp*RuCl(PPh_3_)_2_ and Cp*RuCl(COD) effectively catalyze the polymerization of *tert*‐butyl 4‐azido‐5‐hexynoate (tBuAH), an AB‐type monomer. Increasing the catalyst concentration higher yields were achieved. Specifically, at a 20 mol% catalyst loading, CpRuCl(PPh_3_)_2_ and CpRuCl(COD) achieved yields of 88% and 67%, respectively—both surpassing the 56% yield obtained through CuAAC polymerization. However, the method is limited by the formation of lower molecular weight polymers (*M*_w_ = 28 000 g·mol^−1^).

**FIGURE 18:**
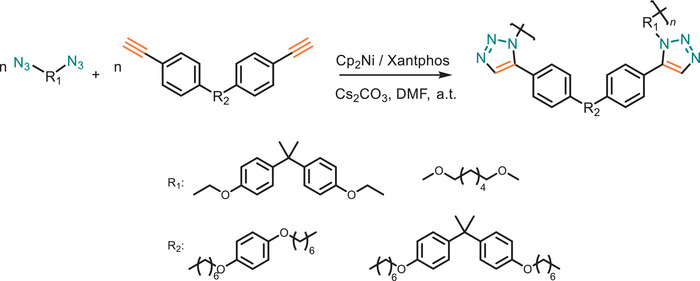
Nickel‐catalyzed polymerization of diazide and tyrine monomers toward 1,5‐regioregular PTAs.

Nickel catalysts are less frequently used in AAC reactions. Interestingly, the regioselectivity in NiAAC can be reversed by employing different nickel catalysts [155, 156, 157, 158, 159]. Recent study from Huang et al. has presented an efficient method for synthesizing 1,5‐regioregular PTAs via NiAAC under mild conditions (Figure 18) [54]. This method uses the Cp_2_Ni/Xantphos/Cs_2_CO_3_ catalytic system to generate high molecular weight PTAs (up to 67 900 g·mol^−1^) in high yields (up to 96.7%) within 30 min at ambient temperature. This approach is a promising addition to sustainable polymer synthesis due to its high efficiency, mild conditions, and use of less toxic materials.

Several reports have demonstrated the use of Ag(I) catalysts for the AAC reaction, operating at ambient temperature and selectively forms 1,4‐disubstituted 1,2,3‐triazole rings [160, 161, 162, 163, 164]. This provides a copper‐free alternative for synthesizing 1,4‐disubstituted triazoles. Additionally, AgAAC prevents side reactions often seen with copper, such as the formation of copper‐azide complexes or alkyne decomposition. Ag(I) catalysts is more tolerant to a wider variety of solvents and functional groups [165] and further can be embodied to the heterogeneous materials [166], offering the advantage of catalyst recyclability, though AgAAC has not yet been applied in polymerization procedures. These findings and availability of Ag, underline that AgAAC could be a valuable tool in polymer chemistry, offering an alternative to traditional copper‐catalyzed methods.

Iridium‐catalyzed azide‐alkyne cycloaddition (IrAAC) is a promising alternative to CuAAC, particularly in terms of lower toxicity and milder reaction conditions [167]. Sun and coworkers reported the cycloaddition of electron rich internal thio alkynes with organic azides in the presence of simple and commercially available [Ir(COD)Cl]_2_ catalyst which resulting trisubstituted triazole ring with absolute regiocontrol [56]. The IrAAC polymerization has been utilized in polymer chemistry for constructing sequence‐defined polytriazoles. A notable study by Wang et al. demonstrated a two‐step iterative strategy involving azidation and IrAAC to synthesize dual sequence‐defined polytriazoles [55]. This approach allowed for the incorporation of diverse functional groups, resulting in uniform tetramers and larger macromolecules with up to 18 side chains and molecular weights exceeding 3900 g·mol^−1^. Additionally, tandem mass spectrometry analysis revealed three major fragmentation patterns, indicating the high decodability of polymers and potential applications in high‐capacity digital polymers. While IrAAC polymerization holds great potential, particularly for precise construction of stereocontrolled oligomers/polymers [57, 58, 59, 60], its cost and availability remain a barrier. Therefore, from a sustainability perspective, it raises significant concerns. Iridium is a scarce and expensive precious metal with limited global availability, primarily sourced as a byproduct of platinum and nickel mining. The extraction and refining processes for iridium are energy‐intensive and environmentally damaging, contributing to its high environmental footprint. Addition to that the recyclability of iridium catalysts presents additional challenges compared to copper catalysts, as some systems may require specialized methods for catalyst recovery and reuse.

As an alternative, Muthusubramanian and coworkers reported the synthesis of triazoles under the heterogeneous catalysis of nanoporous titania‐supported gold nanoparticles ((AuNPs/TiO_2_) in aqueous media [168]. AuNPs/TiO_2_ were synthesized and employed as a recyclable heterogeneous catalyst for the green synthesis of 1,2,3‐triazoles in an aqueous medium. The AuNPs/TiO_2_ catalyst facilitated the cycloaddition between various organic azides and terminal alkynes at room temperature, with the reactions typically reaching completion within 1 h under mild conditions. The nanoporous structure of the titania support provided a high surface area for efficient dispersion of the gold nanoparticles, which in turn promoted rapid mass transfer and high catalytic activity, affording the triazole products in excellent yields and with high regioselectivity. Post‐reaction, the catalyst was recovered and reused over multiple cycles without significant loss of performance. This strategy is compatible with a wide range of terminal alkynes and azides. Even electron‐deficient symmetrical internal alkynes can react with azides to yield fully substituted triazoles under these conditions. Later work from Wang and coworkers, (Au core)@(Cu_2_O shell) nanostructures exhibiting strong plasmonic properties were synthesized and employed to drive click reactions for the regioselective production of 1,2,3‐triazoles [169]. Under visible light irradiation (420–780 nm), the plasmonic excitation of the Au core generated hot holes that effectively activated the azide and alkyne reactants, facilitating a rapid and regioselective cycloaddition under mild and ambient temperature conditions. The process bypassed the need for traditional copper catalysts, aligning well with sustainable and Green Chemistry principles by minimizing hazardous reagents and energy input. Detailed mechanistic studies confirmed that the hot hole transfer from the nanostructures played a pivotal role in steering the reaction pathway toward the selective formation of 1,4‐disubstituted triazoles. Moreover, the robust nature and reusability of the (Au core)@(Cu_2_O shell) catalyst further underscore its potential for scalable applications in organic synthesis. Even though AuAAC is a powerful tool in organic synthesis [170], while offering advantages in terms of functional group tolerance, the high cost of gold is the challenge that needs to be addressed.

Zinc acetate (Zn(OAc)_2_) also holds promising potential in AAC reaction in water, especially catalysing the reaction between azide and internal alkyne [171]. ZnAAC also represents a more sustainable alternative compared to the CuAAC or IrAAC due to the relative abundance in the Earth`s crust and comparable lower toxicity. Zinc is widely available, cost‐effective, and easier to extract compared to many other metals, making it an attractive choice for Green Chemistry. Its catalytic activity in azide‐alkyne cycloaddition offers efficient and mild reaction conditions, reducing energy consumption and aligning with sustainable practices. However, challenges remain, including the need for high catalyst loadings in some reactions and potential limitations in catalyst recyclability, which can generate waste over multiple cycles. Additionally, while zinc is more abundant, its widespread use in various industries (e.g., galvanization, batteries) could lead to localized supply pressures if not managed responsibly (Figure 15). Innovations in zinc catalyst design, such as immobilization on reusable supports, could further enhance its sustainability by improving efficiency and recyclability.

### 4.5 Metal‐Free AAC Reactions (MFAAC)

When discussing metal‐free click polymerization, the focus often shifts to thermal or strain‐promoted click reactions. In this chapter, we will explore an alternative approach—metal free organocatalytic click polymerization—demonstrating how polymerization can be achieved without the use of transition metals. The benefits of organocatalyzed systems, particularly their sustainability, operational simplicity, and compatibility with biocompatible and green processes, make them a compelling alternative to traditional metal‐based catalysis. With continued advancements, they hold great promise for a wide range of applications in academic, industrial, and environmental chemistry [172]. In this direction, Bonacorso and coworkers have introduced a simple approach to obtain 4‐acyl‐1‐substituted‐1,2,3‐triazole in excellent yields by employing a deep eutectic solvent composed of chloroform and ethylene glycol (1:2) as green reaction media [173]. This method offers several advantages for polymer chemistry, including easy workup, metal‐free conditions, cost‐effectiveness, biodegradability, and reusability without any significant loss in yield. However, it has not been used in any polymer synthesis yet.

**FIGURE 19:**
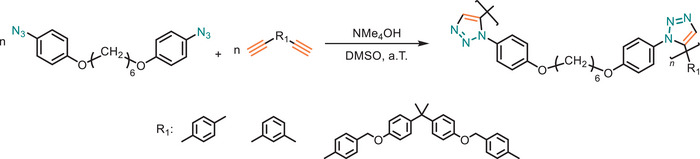
Metal‐free organocatalytic NMe_4_OH‐mediated polymerization under ambient temperature, which eliminating metal contamination and waste.

In 2015, Tang and his team has suggested alternative catalytic methods for the synthesis of 1,5‐regioregular PTAs. In the study tetramethylammonium hydroxide (NMe_4_OH) has been used in the click polymerization in DMSO at ambient temperature and 1,5‐regioregular PTAs with *M*_w_ up to 56 000 g·mol^−1^ were obtained in high yields (up to 96%) (Figure 19) [63]. The base‐catalyzed approach aligns with green chemistry principles by eliminating metal catalysts, reducing environmental toxicity, and avoiding purification challenges typically associated with metal residues. Despite these advantages, the process presents certain limitations. The reliance on a strong base introduces potential substrate compatibility issues, particularly for molecules with base‐sensitive functional groups.

### 4.6 Strain‐Promoted Azide‐Alkyne Cycloaddition Reaction

The strain‐promoted azide–alkyne cycloaddition (SPAAC), introduced by Carolyn Bertozzi and colleagues in 2004, represents a significant advancement in click chemistry. aligns closely with Green Chemistry and Sustainability principles by eliminating the need for metal catalysts, reducing toxic waste, and enabling biocompatible and energy‐efficient polymerization. Unlike copper‐catalyzed reactions, SPAAC avoids potential metal contamination, making it ideal for biomedical applications and environmentally friendly material synthesis. Additionally, its mild reaction conditions reduce energy consumption, and the use of renewable or bio‐derived cycloalkynes further enhances its sustainability, making SPAAC a promising approach for eco‐conscious polymer and bioconjugation strategies [64].

According to classical theory, achieving high molecular weight polymers via homogeneous step‐growth polymerization requires strict stoichiometric balance between AA and BB monomers. Even a minor imbalance can severely reduce the molecular weight, leading to diminished mechanical properties and limited practical applications. To overcome this limitation, researchers have developed stoichiometric imbalance‐promoted polymerization methods that eliminate the equal reactivity of functional groups. A common feature of these approaches is the incorporation of a *self‐accelerating* condensation reaction, which enables the formation of high molecular weight polymers despite inherent stoichiometric challenges [174, 175, 176].

**FIGURE 20:**
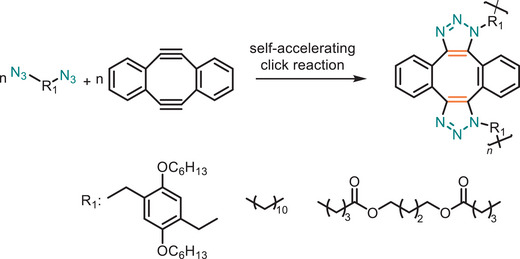
Stoichiometric imbalance‐promoted homogeneous step polymerization based on DSPAAC click chemistry.

Zhang and coworkers first reported a self‐accelerating click reaction based on double‐strain‐promoted azide–alkyne cycloaddition (DSPAAC), marking its inaugural use in step polymerization (Figure 20) [65]. This innovative approach introduced a novel stoichiometric imbalance‐promoted homogeneous step polymerization method using *sym*‐dibenzo‐1,5‐cyclooctadiene‐3,7‐diyne (DIBOD) and various bis‐azide compounds (N_3_‐R‐N_3_) as respective monomers. A key advantage of this DSPAAC‐based polymerization lies in its self‐accelerating nature, which enables the formation of high molecular weight polymers even under stoichiometric imbalance conditions—a limitation in conventional step‐growth polymerizations. The self‐accelerating property of DSPAAC is also a significant advantage as contributes to lower energy consumption during the polymerization process, adhering to the Design for Energy Efficiency principle of Green Chemistry. By using an excess molar ratio of DIBOD to bis‐azide monomers, the polymerization reaction proceeds efficiently, overcoming kinetic barriers typically encountered in step polymerization.

Zhang and colleagues later introduced a novel approach for synthesizing cyclic polymers using a UV‐induced SPAAC reaction, which aligns with Green Chemistry principles by offering an energy‐efficient, catalyst‐free alternative to traditional polymerization methods [177]. This light‐triggered process has reduced the need for harsh chemical reagents or elevated temperatures, which aligns with the Design for Energy Efficiency principle. Still the proces introduces an energy input that may not be fully aligned with sustainability goals when considering the broader environmental impact. Moreover, the need for UV light activation, often generated from non‐renewable energy sources, remains a limitation in terms of Green Chemistry. The reaction allows for precise control over the polymer topology, enabling the synthesis of tailored macromolecular structures. This aspect supports the Green Chemistry principles of atom economy and waste reduction, as it ensures that most of the reactants are efficiently incorporated into the polymer products without the formation of significant by‐products.

In parallel, Dong and colleagues harnessed the intrinsic reactivity of strained alkynes and azides to construct giant polymeric chains via SPAAC, a catalyst‐free process, further reinforcing the advantages of Green Chemistry in polymer synthesis [178]. This method also relies on the high strain energy of cycloalkynes to drive the formation of 1,2,3‐triazole linkages, bypassing the need for metal catalysts, which are often associated with environmental and health concerns. By achieving this transformation under ambient conditions, the approach minimizes the energy input required, offering a sustainable pathway for high molecular weight polymers. Moreover, the operational simplicity and robust performance of the reaction further support its practicality for industrial‐scale applications.

These works collectively demonstrate the transformative potential of SPAAC in advancing sustainable polymer chemistry. By offering catalyst‐free, energy‐efficient, and precisely controlled polymerization strategies, SPAAC facilitates the construction of a wide range of polymeric structures that adhere to green chemistry principles such as energy efficiency, waste reduction, and biocompatibility. However, challenges remain, particularly in the design of alkyne monomers, which require careful consideration of their reactivity, availability, and sustainability. Continued progress in these areas will be crucial for further improving the environmental compatibility of SPAAC‐based polymer synthesis and ensuring its broader application in sustainable material production.

## 5 Conclusions and Outlook

Click polymerization has emerged as a transformative tool in macromolecular synthesis, offering unparalleled efficiency, selectivity, and modularity. The azide‐alkyne cycloaddition (AAC), particularly in its copper‐catalyzed (CuAAC) and strain‐promoted (SPAAC) forms, has played a pivotal role in advancing polymer chemistry, enabling the development of well‐defined architectures with tailored functionalities. However, as sustainability becomes an increasingly pressing priority, it is crucial to critically assess and optimize click polymerization methodologies to align with Green Chemistry principles and circular economy models.

This mini‐review has highlighted the sustainability potential and challenges of AAC‐based polymerization, with a focus on catalyst choice, reaction conditions, solvent use, and end‐of‐life strategies for materials. While CuAAC remains the gold standard due to its high efficiency and regioselectivity, concerns over copper toxicity, metal residue contamination, and energy consumption necessitate the development of greener alternatives. Metal‐free and bio‐based approaches, such as organocatalytic systems, solvent‐free reactions, and enzymatic synthesis of azides, offer promising pathways toward more sustainable click polymerization. The growing interest in recyclable catalysts, heterogeneous catalysis, and light‐driven polymerization further demonstrates the commitment of the field to reducing waste and improving energy efficiency. For example, conducting the azide‐alkyne cycloaddition reaction—a key click chemistry process—in pure water at ambient temperature and under aerobic conditions has demonstrated notable sustainability benefits. These include eliminating the need for organic solvents, reducing energy consumption, and simplifying reaction conditions. However, challenges persist. The limited solubility of reactants in water can hinder reaction efficiency, slower reaction kinetics may necessitate longer reaction times, and side reactions, such as hydrolysis, can compromise polymer purity. Additionally, while water is considered as a green solvent, its large‐scale use raises concerns about availability and sustainability, particularly in water‐scarce regions.

Thus, while click polymerization methodologies represent a significant step toward greener and more sustainable polymer chemistry, further optimization is essential to overcome these challenges. Efforts must focus on improving water‐compatible reactants, designing catalysts with higher activity and selectivity, and precise control over the resulting materials. Through such advancements, click chemistry has the potential to enhance both the environmental friendliness and the practicality of polymer synthesis, paving the way for broader applications in sustainable materials development. Thus, while click chemistry approach enhances environmental friendliness, optimization is required to address these challenges. Therefore, several key challenges must be addressed to fully integrate click polymerization into the next generation of sustainable materials science

- **Catalytic Systems**: The development of catalysts derived from abundant and renewable resources, such as biocompatible organocatalysts or recyclable heterogeneous catalysts with high efficiency and reusability to reduce waste. Future research should also focus on minimizing or eliminating the use of toxic or rare metals, opting for earth‐abundant alternatives like zinc or iron where applicable, or enzymatic alternatives.
- **Reaction Conditions**: The transition towards more milder conditions, such as ambient temperature and pressure, solvent‐free, light‐induced, and/or microwave‐assisted transformations, is essential to minimize energy consumption and carbon footprints. Alternatively, in order to ensure improving energy efficiency and reducing potential degradation of sensitive substrates, it is of crucial importance to optimize reaction kinetics in order to minimize reaction time.
- **Choice of Starting Materials**: The integration of starting materials and monomers from non‐food based renewable, upcycled or biodegradable sources are critical to reduce dependence on petroleum‐derived chemicals and ensure to align with circular economy principles.
- **End‐of‐Life Considerations**: In order to improve polymeric materials longevity and reduce waste generation, it will be essential to design polymers with self‐healing, recyclable or fully degradable functionalities. The way to circular polymer design could be paved by innovations in programmable depolymerisation, enzymatic degradation and upcycling strategies.
- **Scalability and Industrial Translation**: Many sustainable methods of click polymerization are still limited to the laboratory scale. To facilitate their adoption in large‐scale manufacturing and commercial applications, future efforts should prioritize green engineering approaches and life‐cycle assessment (LCA).

In other words, the future of click polymerization lies at the intersection of Green Chemistry, catalysis, and advanced macromolecular engineering. Innovations in catalyst design, energy‐efficient synthesis, and bio‐based materials will reshape the field, driving the development of next‐generation sustainable polymers. Moreover, emerging technologies such as AI‐driven reaction optimization, enzymatic catalysis, and 3D printing of click‐functionalized polymers will further expand the possibilities of tailor‐made, eco‐friendly materials.

Interdisciplinary collaboration between chemists, materials scientists and engineers will be essential to achieve true sustainability. Click polymerization will continue to evolve as a pillar of sustainable polymer chemistry through the integration of LCA, green process engineering, and circular economy models. Thus, these innovations will enable click polymerization to overcome its current limitations and become an important part of the future of high performance, eco‐friendly materials.

## Conflicts of Interest

The authors declare no conflicts of interest.

## Supporting information




**Supporting file**: marc202500171‐sup‐0001‐SuppMat.docx.

## Data Availability

The data that support the findings of this study are available from the corresponding author upon reasonable request.
