# Supplementary material for: Green and Sustainable Chemistry Approaches on Azide‐Based Click Reactions in Polymer Science
Source: Macromol Rapid Commun. 2025 Aug 6;46(23):e00171. doi: 10.1002/marc.202500171 (PMC12687705; doi:10.1002/marc.202500171)
Supplement: Supplementary file 1 — Supporting file: marc202500171‐sup‐0001‐SuppMat.docx. [file MARC-46-e00171-s001.docx]

**Supporting Information**

**Green and Sustainable Chemistry Approaches on Azide-based Click Reactions in Polymer Science**

Hatice Mutlu, C. Remzi Becer, Bercis Pektas, and Azra Kocaarslan^*^

Bercis Pektas

Institut de Science des Matériaux de Mulhouse, UMR 7361 CNRS/Université de Haute Alsace, 15 Rue Jean Starcky, Mulhouse Cedex 68057, France

Rheinland-Pfälzische Technische Universität Kaiserslautern-Landau, Erwin-Schrödinger-Strasse 52, 67663, Kaiserslautern, Germany

Prof. Dr. Hatice Mutlu

Institut de Science des Matériaux de Mulhouse, UMR 7361 CNRS/Université de Haute Alsace, 15 Rue Jean Starcky, Mulhouse Cedex 68057, France

Rheinland-Pfälzische Technische Universität Kaiserslautern-Landau, Erwin-Schrödinger-Strasse 52, 67663, Kaiserslautern, Germany

Leibniz-Institut für Verbundwerkstoffe GmbH (IVW), Erwin-Schrödinger-Straße 58, 67663 Kaiserslautern, Germany

Prof. Dr. C. Remzi Becer

Department of Chemistry, University of Warwick Coventry, CV4 7AL, United Kingdom

Dr. Azra Kocaarslan

Institute of Nanotechnology, Karlsruhe Institute of Technology, Hermann-von-Helmholtz-Platz 1, 76344 Eggenstein-Leopoldshafen, Germany

Institute of Functional Interfaces, Karlsruhe Institute of Technology, Hermann-von-Helmholtz-Platz 1, 76344 Eggenstein-Leopoldshafen, Germany

**Content**

**Table S1.** Features of click chemistry for azide-alkyne cycloaddition (AAC) reaction and their correspondence to the 12 principles of green chemistry and 6 matrices of sustainable chemistry.

| Green Principles | Prevention | Atom Economy | | Less Hazardous Chemical Synthesis | | Designing Safer Chemicals | Safer Solvents and Auxiliaries. | | Design for Energy Efficiency. | Use of Renewable Feedstocks. | | Reduce Derivatives | | Catalysis. | Design for Degradation | | Real-time Analysis | | Accident Prevention | **Evaluation Score** |
| --- | --- | --- | --- | --- | --- | --- | --- | --- | --- | --- | --- | --- | --- | --- | --- | --- | --- | --- | --- | --- |
| Sustainable Matrices | Use renewable feedstocks | | | Use less net water and non-renewable energy | | | Emit less greenhouse gases | | | Produce less waste | | | | Have a smaller carbon footprint | | | Have a facile end-life | | |  |
| Features of click reaction  Type of Activation in AAC | Catalytic System | | | | | | | Conditions | | | | | | | | Material | | | |  |
|  | Ppm loading | | Catalyst Recycling | | Temporal/  spatial control | | | Greener Solvents or Bulk | | | Monomer Availability | | Reaction Time | | | Isomerization control | | Solubility | |  |
| Thermal Activated (TAAC) | 3 | | 3 | | 2 | | | 3 | | | 3 | | 2 | | | 2 | | 2 | |  |
| Cu-catalyzed (CuAAC) | 3 | | 3 | | 3 | | | 3 | | | 3 | | 3 | | | 2 | | 2 | |  |
| Metal-catalyzed (MAAC) | 2 | | 2 | | 3 | | | 2 | | | 3 | | 3 | | | 2 | | 3 | |  |
| Metal-free (MFAAC) | 1 | | 1 | | 1 | | | 2 | | | 3 | | 2 | | | 2 | | 3 | |  |
| Strain-promoted (DSPAAC) | 3 | | 3 | | 3 | | | 2 | | | 1 | | 2 | | | 1 | | 2 | |  |

**Table S1.** Features of click chemistry for azide-alkyne cycloaddition (AAC) reaction and their correspondence to the 12 principles of green chemistry and 6 matrices of sustainable chemistry. The rating scale ranges from 1 to 3, where 1 indicates "underdeveloped," 2 represents "moderately developed," and 3 corresponds to "well-developed” features in AAC click polymerization processes.
